# Comprehensive characterization and optimization of anti-LRRK2 (leucine-rich repeat kinase 2) monoclonal antibodies

**DOI:** 10.1042/BJ20121742

**Published:** 2013-06-13

**Authors:** Paul Davies, Kelly M. Hinkle, Nour N. Sukar, Bryan Sepulveda, Roxana Mesias, Geidy Serrano, Dario R. Alessi, Thomas G. Beach, Deanna L. Benson, Charles L. White, Rita M. Cowell, Sonal S. Das, Andrew B. West, Heather L. Melrose

**Affiliations:** The Michael J. Fox LRRK2 Monoclonal Antibody Consortium: *MRC Protein Phosphorylation Unit, University of Dundee, Nethergate, Dundee DD1 4HN, Scotland, U.K.; †Department of Neuroscience, Mayo Clinic Jacksonville, 4500 San Pablo Road, Jacksonville, FL 32224, U.S.A.; ‡Department of Neurology, University of Alabama at Birmingham, 1530 3rd Ave S, Birmingham, AL 35294, U.S.A.; §Department of Neuroscience, Mount Sinai School of Medicine, 1425 Madison Ave, New York, NY 10029, U.S.A.; ∥Civin Laboratory for Neuropathology, Banner Sun Health Research Institute, 10515 West Santa Fe Drive, Sun City, AZ 85351, U.S.A.; ¶Department of Pathology, Division of Neuropathology, University of Texas Southwestern Medical Center, 5323 Harry Hines Boulevard, Dallas, TX 75390, U.S.A.; **Department of Psychiatry, University of Alabama at Birmingham, 1530 3rd Ave S, Birmingham, AL 35294, U.S.A.; ††The Michael J. Fox Foundation for Parkinson's Research, Grand Central Station, P.O. Box 4777, New York, NY 10163-4777, U.S.A.

**Keywords:** immunohistochemistry, kinase assay, leucine-rich repeat kinase 2 (LRRK2), Parkinson’s disease, DAB, 3,3′-diaminobenzidine, DMEM, Dulbecco’s modified Eagle’s medium, eGFP, enhanced green fluorescent protein, FBS, fetal bovine serum, GAPDH, glyceraldehyde-3-phosphate dehydrogenase, HEK, human embryonic kidney, KO, knockout, LRRK2, leucine-rich repeat kinase 2, MEF, mouse embryonic fibroblast, MJFF, Michael J. Fox Foundation for Parkinson’s Research, PD, Parkinson’s disease, PEI, polyethyleneimine, PFA, paraformaldehyde, UTSW, University of Texas Southwestern, VDAC, voltage-dependent anion channel, WT, wild-type

## Abstract

Missense mutations in *LRRK2* (leucine-rich repeat kinase 2) are a major cause of PD (Parkinson's disease). Several antibodies against LRRK2 have been developed, but results using these polyclonal antibodies have varied widely leading to conflicting conclusions. To address this challenge, the Michael J. Fox Foundation for Parkinson's Research generated a number of monoclonal antibodies targeting epitopes across the LRRK2 protein. In the present paper, we report optimized protocols and results for ten monoclonal antibodies for immunoblotting, immunohistochemistry, immunoprecipitation and kinase activity assays, in rat, mouse and human brain tissue. Several efficacious antibodies were identified, but results demonstrate that the mouse monoclonal N241A/34 is suitable for most applications, with the best overall rabbit monoclonal antibody being c41-2. These antibodies produced a dominant band of the expected size via immunoblotting and a lack of labelling in tissue derived from *LRRK2*-knockout animals under optimized conditions. A significant proportion of LRRK2 protein localizes to insoluble fractions and no evidence of truncated LRRK2 protein was detected in any fraction from rodent or human tissues. An assay was developed for the robust detection of LRRK2 kinase activity directly from frozen mouse and human brain tissue, but precipitous declines in activity were observed that corresponded to increasing post-mortem intervals and processing times. Finally, we demonstrate the highest levels of brain-localized LRRK2 in the striatum, but note differential expression patterns between rat and mouse in both striatum and cortex. Anti-LRRK2 monoclonal antibodies that are unlimited in availability together with the proposed standardized protocols should aid in the definition of LRRK2 function in both health and disease.

## INTRODUCTION

Rare missense mutations and more common genetic variability in the *LRRK2* (leucine-rich repeat kinase 2) gene are known to be important susceptibility factors to late-onset typical PD (Parkinson's disease) [1]. The LRRK2 protein is a large multidomain enzyme with both kinase activity and GTPase activity. The physiological cellular function of LRRK2 is not clear despite strong evolutionary conservation of this class of protein. Some evidence suggests possible functions in neurite outgrowth, vesicular trafficking, protein translation, autophagy, neurotransmitter release and neuroinflammation (reviewed by [2,3]). However, the pathogenic mechanisms and pathways underlying LRRK2 function in disease are not fully understood. *In vitro* data evaluating LRRK2 pathogenic mutations generally point towards a gain of function caused by enhanced activity [4,5]. Thus further investigation into LRRK2 may provide insight into pathways and mechanisms that are important in late-onset PD-related neurodegeneration.

LRRK2 protein is thought to be poorly expressed in the mammalian brain relative to most well-characterized protein kinases, and many studies are conflicting in both the biochemical nature of endogenous LRRK2 and the localization of protein in tissue. Central to this problem, polyclonal antibodies that have not been tested in KO (knockout) animals have formed the vast basis of the available literature. Our previous work demonstrated that commercially generated antibodies, available at the time, lacked significant specificity and sensitivity for reliable detection of LRRK2 protein [6–8]. Although *LRRK2* mouse and rat KO animals are now available, most lots of these initial polyclonal antibodies are no longer obtainable leaving past work open to interpretation. In addition, kinase assays have not been reported for assessment of activity of endogenous LRRK2 in brain tissue.

Recent efforts have been placed on developing and characterizing renewable (i.e. monoclonal) antibodies with more selectivity and sensitivity for LRRK2 detection and purification, many of which were developed with support from the MJFF (Michael J. Fox Foundation for Parkinson's Research). In the present study, we utilized these renewable anti-LRRK2 monoclonal antibodies with the most informative control tissues to develop robust standardized protocols. We were successful in identifying the most specific antibodies for deployment in mouse, rat and human tissues. Techniques detailed in the present paper include immunoblotting, immunocytochemistry, immunohistochemistry and immunoprecipitation, and the results from each protocol were reproduced in multiple laboratories within the Consortium to ensure utility to other laboratories. Our results are expected to facilitate numerous future studies investigating the role of LRRK2 in health and disease.

## EXPERIMENTAL

Dozens of variations of protocol were attempted for each technique for optimization of the signal to noise ratio, with each attempt informing the next variation. For space considerations, only the most robust and reliable protocols that have worked consistently in multiple laboratories are detailed below. Detailed protocols of all these techniques can be found on the Michael J. Fox Foundation website (http://www.michaeljfox.org/research/research-tools.html). It should be noted that batch to batch variation, namely effective concentrations compared with reported titres, was observed with some of the antibodies, thus an initial titration is recommended when reproducing these protocols.

### Animals

All animal protocols were approved by the authors’ respective Institutional Animal Care and Use Committee (or equivalent ethical review panel) and were in accordance with either the National Institute of Health Guide for the Care and Use of Laboratory Animals (NIH Publications No. 80-23) revised 1996 or with U.K. Home Office Animals (Scientific Procedures) Act 1986. Two different *LRRK2*-KO lines were used: *LRRK2* exon 2-deleted mice on a background of C57BL/6 were developed by Huabin Cai and co-workers [9] and obtained directly from Dr Cai or via Jackson Laboratories; and *LRRK2* exon 41-deleted mice on a C57BL/6J were developed by Melrose and Farrer and co-workers [10]. Both strains were found to have undetectable LRRK2 protein and no truncated LRRK2 products were detected by immunoblotting. Mice were genotyped using routine PCR. *LRRK2*-KO rats on a Long Evans Hooded background were developed by Sigma Advanced Genetic Engineering Labs, via targeted genomic editing to introduce a 10-bp deletion in exon 10 via Zinc-Finger Nuclease technology. This deletion results in an early frameshift and the resultant transcript is degraded by nonsense-mediated decay (results not shown). Accordingly, the KO rats were found to have undetectable full-length LRRK2 protein and no truncated LRRK2 products that could be detected by immunoblotting.

### Human tissue

Human brain tissue from neurologically normal adults was obtained from diagnostic autopsies performed at the University of Texas Southwestern (UTSW) Medical Center, from the Brain and Body Donation Program at Banner Sun Health Research Institute (BSHRI), Sun City, AZ, U.S.A., and from the National Institute of Child Health and Human Development Brain and Tissue Bank (NICHD BTB), Baltimore, MD, U.S.A.

### Antibodies

**Figure 1:**
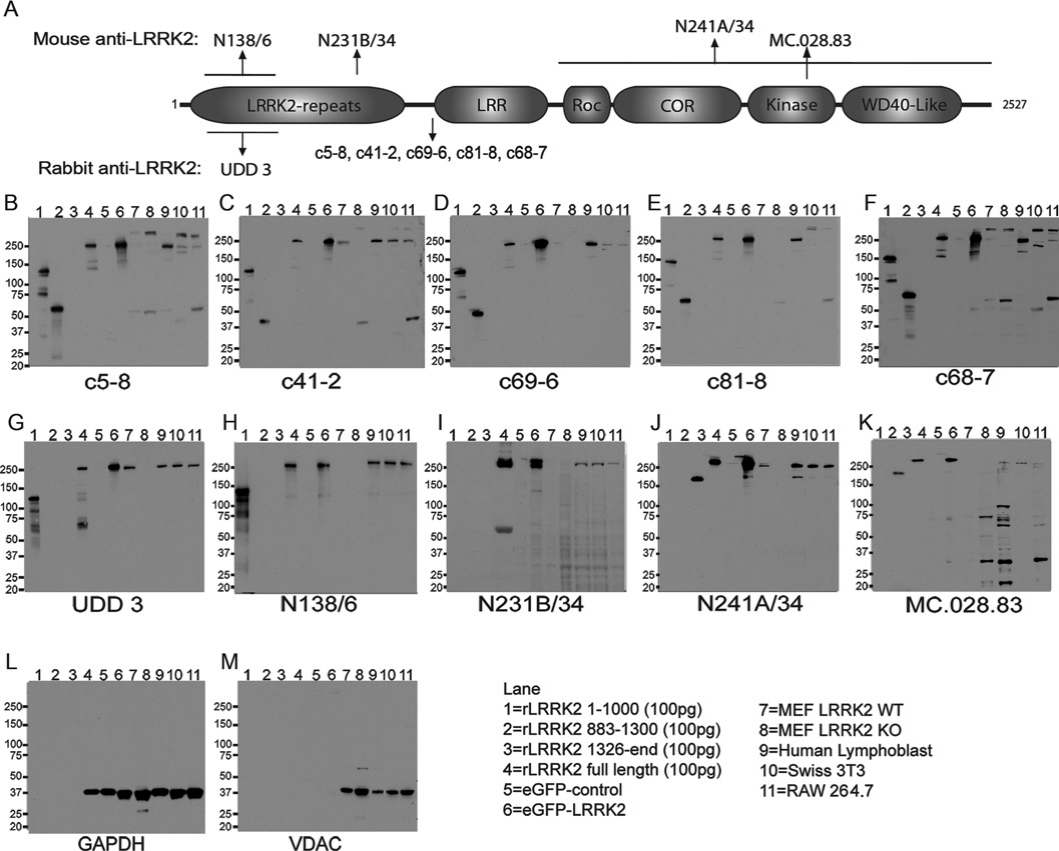
Epitope mapping and detection of recombinant and endogenous LRRK2 in cell lines (**A**) Schematic representation of the LRRK2 protein domain structure, indicating the approximate epitope site for each of the ten monoclonal antibodies tested. LRR, leucine-rich repeat domain; Roc, Ras of complex proteins; COR, C-terminal of Roc. (**B**–**M**) Summary blots of anti-LRRK2 antibodies against various bacterial and mammalian expressed human recombinant LRRK2 and endogenous mouse and human LRRK2. Primary antibodies were used at a concentration of 1 μg/ml except for UDD 3, which was used at 0.1 μg/ml. GAPDH and VDAC blots are included as loading controls. rLRRK2, recombinant LRRK2. Molecular masses are indicated in kDa.

Rabbit monoclonal antibodies against LRRK2 were purchased from Epitomics and are as follows: c5-8, c41-2, c69-6, c81-8, c68-7 (developed by the MJFF) and UDD 3 (developed by Professor Dario Alessi's group at the University of Dundee). Mouse monoclonal antibodies against LRRK2 were purchased from Covance (MC.028.83, developed by Dr Justus Dächsel at Mayo Clinic Jacksonville in collaboration with the MJFF) and University of California Davis/NIH (National Institutes of Health) NeuroMab Facility (N241A/34/34, N138/6 and N231B/34). Figure 1(A) shows the epitope location for each antibody. Isotype IgG controls were purchased from the respective vendors (as above) or from Sigma for selected experiments.

### Cell-line culture

*LRRK2*-KO [9] and WT (wild-type) MEFs (mouse embryonic fibroblasts) were isolated and immortalized by Dr Francisco Inesta-Vaquera at the MRC (Medical Research Council) Protein Phosphorylation Unit, Dundee. Human lymphoblasts, WT and KO MEFs and Swiss 3T3 cells were grown to 90% confluency in DMEM (Dulbecco's modified Eagle's medium) supplemented with 10% (v/v) FBS (fetal bovine serum), 1% (w/v) non-essential amino acids, 1% (v/v) 100× L-glutathione and 1% (v/v) 100× sodium pyruvate. HEK (human embryonic kidney)-293T cells were grown in DMEM supplemented with 10% (v/v) FBS, 1% (v/v) 100× L-glutamine and transfected using the PEI (polyethyleneimine) method [11]. RAW264.7 cells were grown in DMEM containing 10% (v/v) heat-inactivated FBS and 1% (w/v) penicillin/streptomycin. DNA–PEI complexes were allowed to form in serum-free medium for 20 min before being added dropwise to each dish [human eGFP (enhanced green fluorescent protein)–LRRK2, plasmid number DU19169 (Division of Signal Transduction Therapy, University of Dundee) 60 μl, 20 μg of DNA, 1 mg/ml PEI]. Protein expression was allowed to continue for 36 h before lysis. For adherent cells, lysis buffer [50 mM Tris/HCl (pH 7.5), 1 mM EGTA, 1 mM EDTA, 1% (w/v) sodium orthovandate, 10 mM sodium glycerophosphate, 50 mM NaF, 5 mM sodium pyrophosphate, 270 mM sucrose, 1 mM benzamide, 2 mM PMSF and 1% (w/v) Triton X-100] was added directly to the culture dish before scraping. Suspension cells were collected and resuspended in lysis buffer. Cells were then homogenized on ice in 1-s bursts in 10-fold (v/w) excess of lysis buffer. Lysates were clarified for 20 min at 20000 ***g*** at 4°C, and supernatant was collected and quantified using the Bradford method [12].

### Immunoprecipitation

Antibodies were coupled to either Protein A (rabbit primary) or Protein G (mouse primary) –Sepharose beads at a 1 μg:1 μl bead ratio by incubation at 4°C with rotation. Following incubation, beads were centrifuged at 13000 ***g*** for 1 min, the supernatant was removed, and the beads were washed four times in PBS to remove unbound antibody. Clarified cell lysates were incubated at a ratio of 10 ml of antibody-bound beads to 1 mg of total protein for overexpressed material, 5 mg for endogenous cellular material or 10 mg for brain tissues, for 1 h at 4°C. The complexes were then collected by centrifugation at 13000 ***g*** for 2 min before repeated washing (three times) in lysis buffer plus 500 mM NaCl. For direct elution, 4× LDS (lithium dodecyl sulfate) loading buffer was then added to the beads and the mixture was incubated at 100°C for 10 min. The eluent was then diluted 2-fold and collected by centrifugation through a 0.22 μm pore-size Spinex column. 2-Mercaptoethanol was then added to the eluent to 1% and the sample was incubated for 5 min at 70°C before immunoblotting. For kinase assays, the beads were resuspended in buffer A [50 mM Tris/HCl (pH 7.5), 1 mM EGTA, 270 mM sucrose, 1 mM benzamide and 2 mM PMSF].

### Kinase assays

Immunoprecipitates were prepared as described above. Protein (1 mg) was used for cells lines expressing human recombinant LRRK2, 5 mg for cell lines expressing endogenous LRRK2 and 10 mg for tissue per 10 μl of antibody-bound beads. Tissues used were mouse whole brain, mouse whole kidney and human cerebellum. Peptide kinase assays were set up in a total volume of 50 μl with immunoprecipitated LRRK2 in 50 mM Tris/HCl (pH 7.5), 0.1 mM EGTA, 10 mM MgCl_2_ and 0.1 mM [γ-^32^P]ATP (~300–500 c.p.m./pmol, PerkinElmer) in the presence of 20 μM Nictide peptide substrate (RLGWWRFYTLRRARQGNTKQR [13]). Assays were carried out at 30°C for 30 min with shaking. Reactions were terminated by applying 30 μl of the reaction mixture on to P81 phosphocellulose paper and immersing in 50 mM phosphoric acid. After extensive washing, the radioactivity in the reaction products was quantified by Cerenkov counting. Kinase assay data were analysed using a two-way ANOVA, with time and lysate source as factors.

### Rodent and human tissue lysate generation

Male 12-week-old Long–Evans rats, either WT or KO, were deeply anaesthetized with isoflurane and transcardially perfused with 300 ml of room-temperature (25°C) saline. Brains were removed and 200 mg of forebrain was dissected and placed on ice. Male 12-week-old C57BL/6J mice, either WT or *LRRK2*-KO [10], were deeply anaesthetized with isoflurane and transcardially perfused with 50 ml of room-temperature saline under isoflurane anaesthesia. Brains were removed and 100 mg of forebrain was dissected and placed on ice. Tissue was immediately added to 1 ml of PBS (pH 7.4) containing 1× protease inhibitors (Roche) and subjected to ten strokes of Dounce homogenization. The suspension was centrifuged at 20000 ***g*** for 5 min and supernatant combined into Laemmli buffer to create lysate 1. The pellet was resuspended into 1 ml of PBS supplemented to 500 mM NaCl and protease inhibitors and subjected to 15 strokes of Dounce homogenization and the suspension was centrifuged for 20000 ***g*** for 5 min. The supernatant was resuspended in Laemmli buffer (creating lysate 2) and the pellet was resuspended into 1 ml of PBS supplemented to 500 mM NaCl, 1% Triton X-100 and protease inhibitors. The suspension was vortex-mixed for 30 s and subjected to rotation on a wheel for 1 h. The suspension was centrifuged at 20000 ***g*** for 10 min and the supernatant was resuspended in Laemmli buffer, creating lysate 3. The pellet was resuspended in 1 ml of PBS supplemented to 500 mM NaCl, 1% (w/v) SDS and protease inhibitors. The suspension was centrifuged at 20000 ***g*** for 10 min, and the supernatant was resuspended in Laemmli buffer to create lysate 4. Finally, the pellet was resuspended in 1 ml of Laemmli buffer and sonicated for 10 s to create lysate 5 (rat and mouse brain lysate only). As a control, 100 pg of human WT LRRK2 recombinant protein (amino acids 970–2527, Invitrogen) was loaded.

### Immunoblotting

Lysates were loaded directly on to either 4–12% Bis-Tris gels (Invitrogen) or 7.5% TGX™ (Bio-Rad Laboratories) polyacrylamide gels and electrophoresed at 10 V/cm for ~1.5 h. Acrylamide gels of 10% or less are suitable for resolution and transfer of LRRK2 protein; higher percentages impede complete LRRK2 transfer to membranes. SDS/PAGE gels were combined with either activated PVDF or nitrocellulose (Millipore), with equivalent results, into Tris-Glycine transfer buffer overnight at 25 V at 4°C. Membranes were washed, and successful protein transfer and equality of loading were verified with Ponceau S (Sigma) stain. Membranes were blocked in TBS-T (Tris-buffered saline/0.1% Tween 20) with 5% (w/v) non-fat dried skimmed milk powder or 5% BSA (approximately equivalent results were obtained using either BSA or milk powder as a blocking reagent) for 1 h and primary antibody was applied overnight at 4°C or for 1–2 h at room temperature. All Epitomics and NeuroMab antibodies were used at 1 μg/ml, with the exception of UDD 3 that was used at 0.1 μg/ml and Covance antibody that was used at 0.1 μg/ml. Secondary antibodies [donkey anti-(rabbit IgG) and goat anti-(mouse light chain-specific IgG), Jackson ImmunoResearch Laboratories] were used at 0.1 μg/ml for 1–2 h at room temperature. Antibodies against GAPDH (glyceraldehyde-3-phosphate dehydrogenase) (Santa Cruz Biotechnology) and VDAC (voltage-dependent anion channel) (NeuroMab) were used at 0.01 μg/ml for 2 h at room temperature. Membranes were developed with ECL (enhanced chemiluminescence) reagent with exposures that varied between 10 s and 1 min.

### Rodent brain free-floating immunohistochemistry

Three male WT and *LRRK2*-KO [10] mice aged 4 months and two male WT and *LRRK2*-KO rats aged 2 months were terminally anaesthetized with ketamine and xylazine cocktail (120 and 20 mg respectively/kg of body mass for mice, 75 and 10 mg respectively/kg of body mass for rats) and transcardially perfused with 50–100 ml of ice-cold PBS containing 0.025% heparin followed by 50–100 ml of ice-cold 4% (w/v) PFA (paraformaldehyde). Brains were dissected and post-fixed at 4°C for 24 h in 4% PFA, then transferred to 30% (w/v) sucrose in PBS at 4°C for cryopreservation. Once saturated in sucrose, brains were flash-frozen in 2-methylbutane cooled on dry ice and stored at −80°C until sectioning at 40 μm on a sledge microtome (Leica). Fresh sections were cut the day before each experiment, as the signal for LRRK2 greatly diminishes with time, with no detectable LRRK2 protein apparent after 1 month of section storage at −20°C. Free-floating immunohistochemistry was performed in 12 (rat) or 24 (mouse)-well plates. Sections were quenched in 0.3% H_2_O_2_ in 100% methanol for 15 min at 4°C with agitation, followed by washing twice with PBS for 5 min. To enable antigen retrieval, sections were incubated with 10 mM sodium citrate (pH 6.0) containing 0.05% Tween 20 for 30 min at 37°C with agitation. Following two 5-min washes, non-specific sites were blocked by incubating sections for 30 min in 3% (w/v) non-fat dried skimmed milk powder in PBS containing 1% (w/v) Triton X-100 at 4°C with agitation. A second blocking step was performed in 10% (v/v) normal serum (matched to secondary antibody species) in PBS for 30 min at 4°C with agitation. For mouse on mouse staining we also included a mouse IgG blocking step (Vector Laboratories). Next, sections were incubated in primary antibody in 5% (v/v) normal serum/PBS containing 0.15% Triton X-100 for 18–36 h at 4°C with agitation.

‘No primary’ antibody and concentration-matched IgG controls were included in each experiment. Before appropriate secondary antibody application (rat adsorbed anti-mouse secondary antibodies were used for rat brain staining with mouse monoclonal antibodies), sections were washed twice in PBS for 5 min and then placed in secondary antibody in 5% (v/v) normal serum/PBS containing 0.15% Triton X-100 for 18–36 h at 4°C with agitation. The next day, sections were washed twice and incubated with Avidin–Biotin Complex reagent (Vector Laboratories) for 30 min, washed twice again, then developed in DAB (3,3′-diaminobenzidine) substrate (Vector Laboratories) for 2–5 min. The sections were placed in distilled water to terminate the DAB development reaction and mounted with 25% ethanol in PBS on to positively charged glass slides (ThermoFisher). Following at least 3 h of air-drying, the slides were dehydrated in ascending alcohols and three changes of xylene and coverslipped with Permount (ThermoFisher).

We obtained the highest LRRK2 signal over background in immunohistochemistry experiments when freshly sectioned brain material was used the same day as perfusions. LRRK2 signal precipitously declined with storage of brain sections at 4°C or −20°C, with most signal lost after just a few days in both rats and mice, yet background signal was unaffected. Thus LRRK2 is unusually sensitive to high-temperature storage conditions. All attempts were made to stain sections as fresh as possible, although storage of whole-frozen and cryopreserved brains at −80°C had smaller deleterious effects and LRRK2 signal could be recovered from brains stored at −80°C for several weeks to months.

Additional methods are available in the Supplementary Online Data at http://www.biochemj.org/bj/453/bj4530101add.htm.

## RESULTS

### Epitope mapping and detection of recombinant and endogenous LRRK2 in cell lines

Antigens used for the production of anti-LRRK2 monoclonal antibodies include recombinant N-terminal fragments of LRRK2, readily soluble from *Escherichia coli* expression systems, as well as kinase-active fragments of LRRK2 lacking the N-terminus, generated in insect cells, and a short peptide synthesized *in vitro* (see Figure 1A for list of antibodies and epitope positions, mapped in the present study). The resulting monoclonal antibodies detect epitopes that span the entirety of the LRRK2 protein. Ten antibodies in total were tested in the present study.

To investigate the specificity and signal quality of the antibodies under evaluation, immunoblots were carried out using recombinant LRRK2 proteins and cell lines that express LRRK2. Figures 1(B)–1(M) shows the immunoblots for each monoclonal antibody with a range of truncated LRRK2 proteins, full-length recombinant LRRK2 protein, overexpressed GFP-tagged human LRRK2 in HEK-293T cells, and several murine cell lines known to be positive for endogenous LRRK2. The LRRK2 fragments confirm the expected position of the LRRK2 epitopes, and notably refine further the position of five of the Epitomics antibodies (c5-8, c41-2, c69-6, c81-8 and c68-7) that were raised against a ~200 kDa large fragment of LRRK2 (amino acids 970–2527), down to a 31-amino-acid span from 970 to 1000. The N138/6 antibody, raised against synthetic peptide corresponding to amino acids 1–500 and the UDD 3 antibody, raised against amino acids 100–500 both detect the N-terminus, as expected. Neuromab N231B/34, which was raised against a synthetic peptide corresponding to amino acids 841–960 did not detect recombinant LRRK2-(883–1300), suggesting that the epitope for this antibody must lie between amino acids 841 and 883. Finally, the Neuromab antibody N241A/34 and Covance antibody MC.028.83, raised against synthetic peptides corresponding to amino acids 970–2527 and 2069–2087 respectively, both cross-react with the C-terminal half of LRRK2 (from amino acid 1326 to the end).

All of the antibodies robustly detected human LRRK2 from both recombinant sources and lysates from HEK-293T cells overexpressing eGFP-tagged human LRRK2 protein. The six Epitomics rabbit monoclonal antibodies had various levels of success in detecting endogenous mouse LRRK2 from MEFs, Swiss 3T3 cells and RAW264.7 cells. Notably, the c5-8, c81-8 and c68-7 antibodies detect a higher-molecular-mass band that is not deleted in *LRRK2*-KO MEFs, suggesting that these antibodies should be used with caution, since other PAGE conditions may not fully resolve the higher-molecular-mass band with the expected size of LRRK2. The Covance MC.028.83 antibody detected multiple prominent lower-molecular-mass bands. No evidence was observed for truncated LRRK2 species in these cell culture lysates despite antibodies spanning the protein, as only a single band of ~280 kDa is apparent. All antibodies tested detected mouse LRRK2 protein, even though these antibodies were raised against peptides corresponding to human LRRK2.

Overall, our recommendation for cellular lysates are as follows: any of the ten antibodies can be used for recombinant human protein, and the rabbit monoclonal antibodies c41-2 and UDD 3 and the mouse monoclonal antibodies N138/6, N231B/34 and N241A/34 work well for the detection of endogenous LRRK2 in human and mouse cell lysates.

### Immunoprecipitation and LRRK2 activity assays

**Figure 2:**
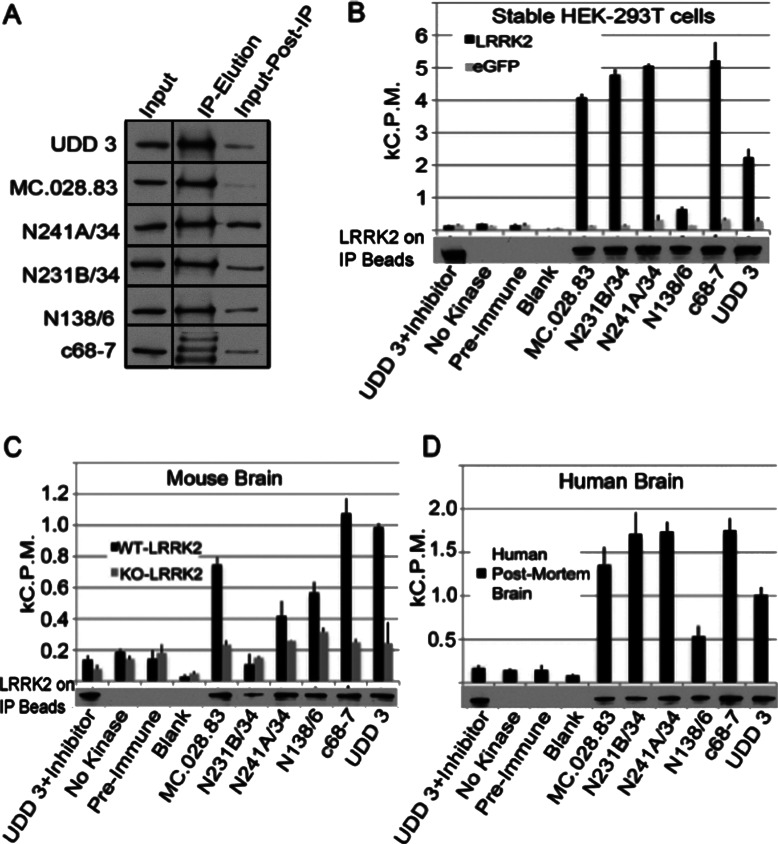
Identification of anti-LRRK2 antibodies suitable for immunoprecipitation and kinase assays in cell lines and brain tissue (**A**) Depletion analysis using the five antibodies assessed to be the most suitable for immunoprecipitation. Immunoprecipitation of LRRK2 was carried out from lysates derived from HEK-293T cells overexpressing human WT LRRK2. For each precipitation, 10 μg of antibody was conjugated to 10 μl of beads. All blots were probed with UDD 3 antibody at 0.1 μg/ml. (**B**–**D**) Assessment of antibody suitability for the immunoprecipitation of LRRK2. All blots were probed with UDD 3 antibody at 0.1 μg/ml and show the LRRK2 on the IP (immunoprecipitation) beads following the kinase assay. Recorded activities were normalized against the LRRK2 IP input, as determined by Western blotting. (**B**) Lysates derived from HEK-293T cells overexpressing human WT LRRK2. For each antibody, 0.5 mg of total lysate was used with 10 μg of antibody to 10 μl of Protein A or G–Sepharose. (**C**) Endogenous LRRK2 mouse brain tissue. LRRK2 was immuoprecipitated from 5 mg of lysates for endogenous cells or 10 mg of lysates from tissue per 10 μl of antibody-bound beads. Relative activity is assessed as counts per minute from ^32^P decay of bound and radiolabelled ATP. (**D**) Endogenous LRRK2 from human tissue. LRRK2 was immuoprecipitated from 10 mg of tissue per 10 μl of antibody-bound beads. Relative activity is assessed as counts per minute from ^32^P decay of bound and radiolabelled ATP.

All of the antibodies were next evaluated for immunoprecipitation from lysates derived from cells overexpressing human LRRK2, mouse brain tissue or human brain tissue. All of the antibodies tested were able to precipitate a proportion of LRRK2 in the whole lysate (results not shown), but the Epitomics rabbit monoclonal antibodies performed relatively less efficiently, with the exception of c68-7 and UDD 3. Despite only a small fraction of total LRRK2 protein removed from the lysate (Figure 2A shows results from the six best antibodies), significant LRRK2 kinase activity was observed from protein bound to the beads. To assess the activity of LRRK2, we performed kinase assays in immunoprecipitates from mouse and human brain tissue using the model substrate Nictide by [γ-^32^P]ATP assay. As a control, each precipitation was carried out in tandem in *LRRK2*-KO MEFs or tissue to show that the activity was dependent on LRRK2 expression. Additionally, the potent LRRK2 inhibitor LRRK2-IN-1 [14] was used for the UDD 3 precipitation to ensure the activity was due to LRRK2 and not to contaminating kinases. Figure 2 shows the activity of precipitated human LRRK2 in HEK-293T cells (Figure 2B), and endogenous LRRK2 from mouse (Figure 2C) and human (Figure 2D) brain. With the exception of N138/6, all of the antibodies precipitated both active human and mouse endogenous LRRK2.

Following on from activity assay optimization, we addressed whether the post-mortem interval affects kinase activity. Tissue from human brain, mouse brain and mouse kidney was incubated on ice for periods of up to 168 h. The tissue was then lysed and LRRK2 was precipitated as above. LRRK2 kinase activity was assessed in the precipitates and compared with recombinant human LRRK2 precipitated from human cells and incubated for the same intervals on ice. Supplementary Figure S1 (at http://www.biochemj.org/bj/453/bj4530101add.htm) plots the decrease in LRRK2 kinase activity over time when tissue was incubated on ice. Activity precipitously declined over time (two-way ANOVA, time as factor, *P*≤0.0001) and the half-life of LRRK2 from both mouse and human tissues was between 2 and 4 h, which was significantly different from recombinant LRRK2 precipitated from HEK-293T cells (two-way ANOVA, lysate source as factor, *P*≤0.0001).

Our recommendations for immunoprecipitation/kinase assays are rabbit monoclonal antibodies c68-7 and UDD 3 in mouse brain and rabbit monoclonal c68-7 and mouse monoclonal antibodies N231B/34 and N241A/34 for human brain.

### Characterization of monoclonal antibody reactivity in mouse brain

#### Immunoblotting

Whole mouse brains from PBS-perfused ~8-week-old WT or *LRRK2*-KO mice were rapidly dissected and protein was extracted in a detergent solubility series (Supplementary Figure S2 at http://www.biochemj.org/bj/453/bj4530101add.htm). This approach allows the resolution of cross-reactive bands under differential extraction conditions. For example, a particular antibody may only react with LRRK2 in salt-soluble fractions, but cross-react with many other proteins in SDS-solubilized fractions. Highly soluble proteins eluted with only mechanical homogenization in PBS (fraction 1) were first removed, followed by proteins solubilized under high-salt extractions, followed by Triton X-100 and then SDS extraction, with subsequent processing in Laemmli buffer. The fractions were processed in equal volumes that were also loaded equivalently into acrylamide gels. The rabbit monoclonal antibodies from Epitomics (Supplementary Figures S2A–S2F) all detected mouse LRRK2 in each of the differential detergent fractions tested in WT mice, although c5-8, c69-6 and c81-8 also detected an equally intense higher-molecular-mass cross-reactive band in both WT and *LRRK2*-KO brain in the SDS-solubilized fraction, consistent with mouse cell lines in Figure 2. c41-2 and c68-7 both detected a band corresponding to LRRK2 in WT, but not *LRRK2*-KO, brain, with much fainter off-target reactive bands apparent with c41-2 in the soluble fractions. c68-7 consistently had a lower affinity or titre compared with other antibodies and, like c41-2, did not cross-react with the high-molecular-mass detergent-solubilized protein even at long exposures. UDD 3 displayed a robust LRRK2 signal in all WT fractions, but substantial lower-molecular-mass bands were also resolved. For the mouse monoclonal antibodies (Supplementary Figures S2G–S2J), Neuromab N138/6 and N241A/34 both produced a single band in the WT with no signal in *LRRK2*-KO that could not be explained by cross-IgG-reactivity (Supplementary Figure S2K). MC.028.83 cross-reacted with lower-molecular-mass bands in addition to secondary antibody cross-reactivity with IgG, which was not removed by perfusion. No specific signal, aside from IgG cross-reactivity, was obtained for N231B/34. No truncated LRRK2 protein was observed in mouse brain lysates.

In summary, for immunoblotting in lysates from mouse brain, we recommend rabbit monoclonal antibodies c41-2 and c68-7.

#### Immunohistochemistry

Previously, we reported an immunohistochemistry protocol using the c41-2 antibody to generate robust signal in WT mice, but not *LRRK2* KO mice [15]. We attempted several additional fixation approaches, antigen-retrieval methods and blocking conditions to optimize the signal to noise ratio. Light fixation of frozen brain sections with 10% acetone completely ablated LRRK2 signal for all antibodies. Similar results were obtained with methanol and gluteraldehyde fixation steps. Importantly, no specific LRRK2 signal has been observed without antigen-retrieval approaches in PFA-fixed brain tissue. It was observed that in rodents where PFA perfusion was not optimal, LRRK2 signal greatly diminished and could not be recovered by post-fixation attempts. Several antigen-retrieval methods were deployed, including low-pH treatment, sodium citrate exposure, steam-incubation and EDTA boiling. A combination of both low pH and sodium citrate was determined to provide the optimal signal to noise ratio, whereas EDTA boiling significantly increased non-specific signal in both KO and WT sections. Finally, we optimized blocking conditions, and tested combinations of Background Buster (Innovex), BSA, non-fat dried skimmed milk powder and non-immune serum. A double blocking protocol of non-fat dried skimmed milk powder and non-immune species-matched serum (secondary antibody) was determined to be optimal.

**Figure 3:**
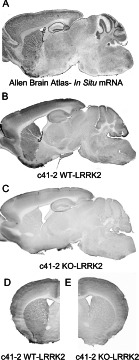
Anatomical distribution of LRRK2 immunostaining in mouse followed a pattern closely resembling that of *LRRK2* mRNA (**A**) mRNA mapped by *in situ* hybridization in the sagittal orientation, taken from the Allen Brain Atlas (http://www.brain-map.org/). (**B** and **C**) Sagittal WT (**B**) and *LRRK2*-KO (**C**) brain sections stained free-floating with Epitomics c42-1 antibody. (**D**) c41-2 staining at the level of the striatum from WT brain and (**E**) *LRRK2*-KO brain in coronal orientation.

**Figure 4:**
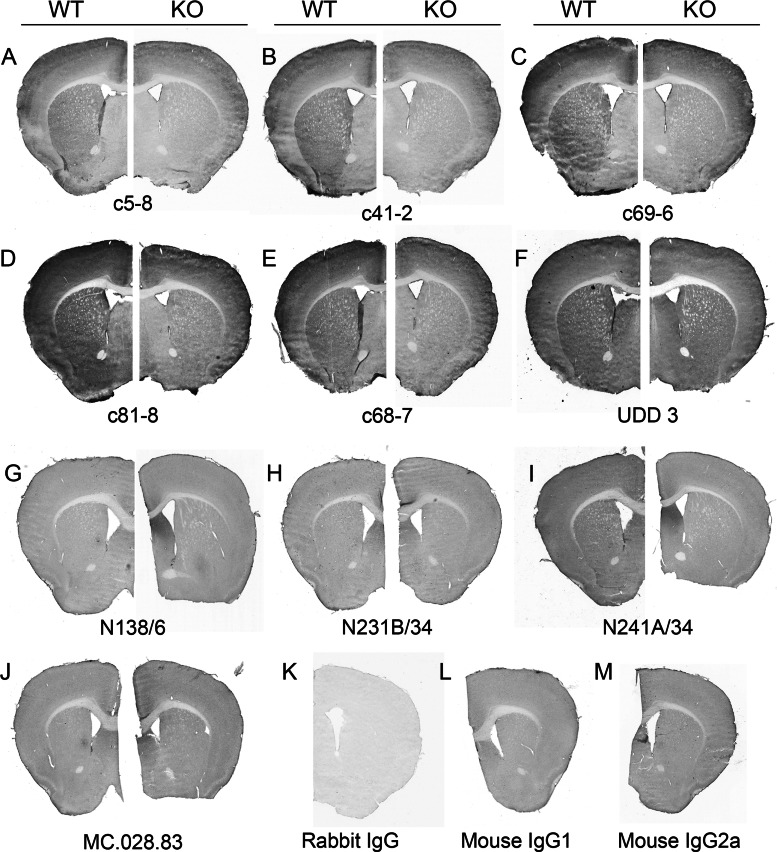
Anti-LRRK2 antibody immunoreactivity in mouse brain (**A**–**J**) Summary of immunostaining for each antibody tested in WT and *LRRK2*-KO mouse brain using PFA-perfused sucrose-preserved brain sections, stained free-floating. Dilutions in μg/ml were as follows: c5-8 (0.05), c41-2 and c69-6 (0.025), c81-8 (0.06), c68-7 (0.5), UDD 3 (0.001), N138/6 (0.13), N231/B/34 and N241A/34 (0.4) and MC.028.83 (0.05). (**K**–**M**) Representative isotype controls for each serotype.

The anatomical distribution of LRRK2 immunostaining in mouse followed a pattern closely resembling that for *LRRK2* mRNA [16–18] (Figure 3). This is best demonstrated in sagittal sections which show the highest *LRRK2* mRNA and protein expression in striatum, cortex and cerebellum. Lesser signal was observed in thalamus and brainstem. When comparing all ten anti-LRRK2 antibodies in WT and *LRRK2*-KO free-floating sections, LRRK2-positive cells were clearly demarcated for the rabbit monoclonal antibodies from Epitomics with the exception of UDD 3 that targets the N-terminal portion of LRRK2 (Figure 4). Little or no specific signal was observed in WT compared with *LRRK2*-KO for the mouse monoclonal antibodies after blocking endogenous IgG. Mouse IgG isotype controls on mouse brain still demonstrated relatively high background due to the concentrations and exposures required to resolve the LRRK2 signal, despite attempts to block this signal. It is possible that conditions required for blocking endogenous mouse IgG reduced LRRK2 antigen accessibility.

In WT mouse, the Epitomics rabbit monoclonal antibodies produced identical staining patterns throughout the cortex and striatum, although with various degrees of intensity. *LRRK2*-KO sections still retained significant colouration with all primary antibody incubations, although this DAB colouration in the *LRRK2*-KO did not correspond to individual cells or features upon inspection at higher magnifications.

Overall, c41-2 produced by far the highest specific signal compared with background noise and thus is our top recommendation for mouse free-floating brain sections.

#### Immunocytochemistry

We extended our studies to dissociated neurons derived from hippocampus of postnatal day 2 mice. Endogenous LRRK2 can be detected in immunoblots at 7 days in culture with increased levels at 21 days in culture (Supplementary Figure S3A at http://www.biochemj.org/bj/453/bj4530101add.htm). Four of the ten antibodies tested for immunocytochemical staining showed a consistent punctate immunostaining pattern in WT neurons that is not present in *LRRK2*-KO neurons (c5-8, c81-8, N231B/34 and N241A/34, arrows, Supplementary Figure S3). Nevertheless, even using the best of four fixation conditions (Supplementary Online Data), the signal is low and requires careful use of controls for interpretation.

### Characterization of monoclonal antibody reactivity in rat brain

#### Immunoblotting

**Figure 5:**
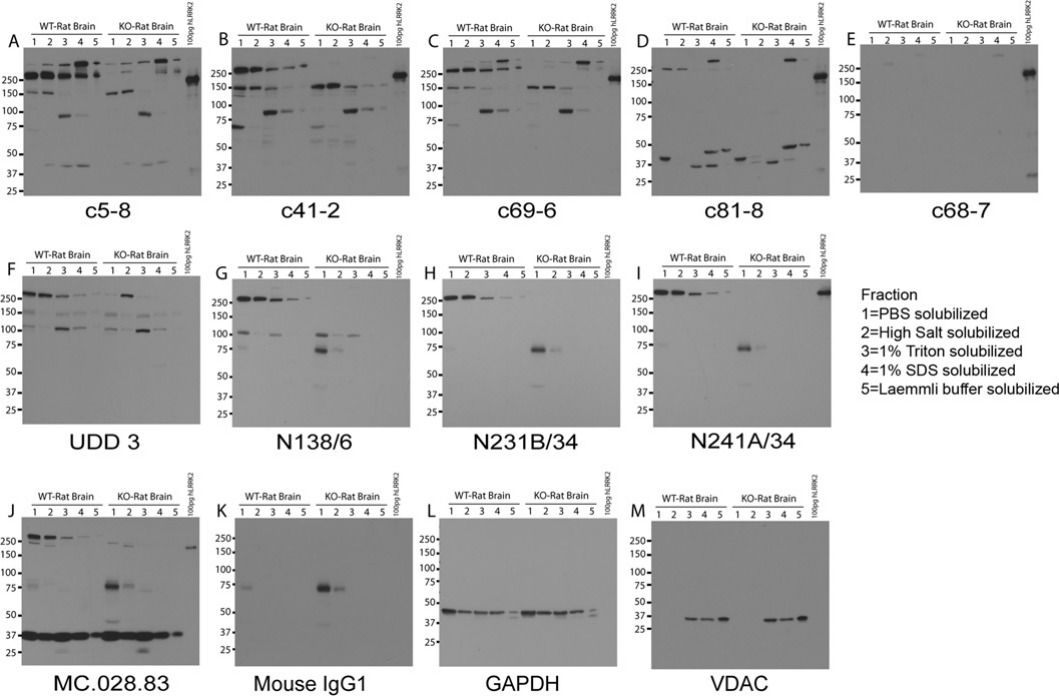
Summary blots of all anti-LRRK2 antibodies tested in WT and *LRRK2*-KO rat brain lysates from a series of differential extractions carried out in equivalent volumes Equal volumes were also loaded on to each gel. A total of 100 pg of human WT LRRK2 protein (amino acids 970–2527) recombinant protein was loaded as a control. All Epitomics and NeuroMab antibodies were used at 1 μg/ml, with the exception of UDD 3 which was used at 0.1 μg/ml, MC.028.83 antibody was used at 0.1 μg/ml. Molecular masses are indicated in kDa.

Lysates derived from whole rat brain solubilized using the conditions described for mouse brain lysates were performed on WT and *LRRK2*-KO rats. Because of significant cross-reactivity with proteins different from the expected size of LRRK2, all rabbit monoclonal antibodies performed poorly for immunoblotting (Figures 5A–5F). Notably, UDD 3 antibody reacted with a protein of size identical with that of LRRK2 that did not diminish in intensity in the *LRRK2*-KO rat. In contrast, all three NeuroMab mouse monoclonal antibodies produced a single LRRK2 band in WT with no significant reactivity against proteins in rat *LRRK2*-KO lysates. In the rat *LRRK2*-KO, some IgG cross-reactivity was observed, even when an anti-mouse secondary antibody pre-absorbed against rat IgG was used. MC.028.83 had a very strong cross-reactive lower-molecular-mass band.

We thus recommend mouse monoclonal antibodies N231B/34 and N241A/34 for detection of rat LRRK2 via immunoblot.

#### Immunohistochemistry

**Figure 6:**
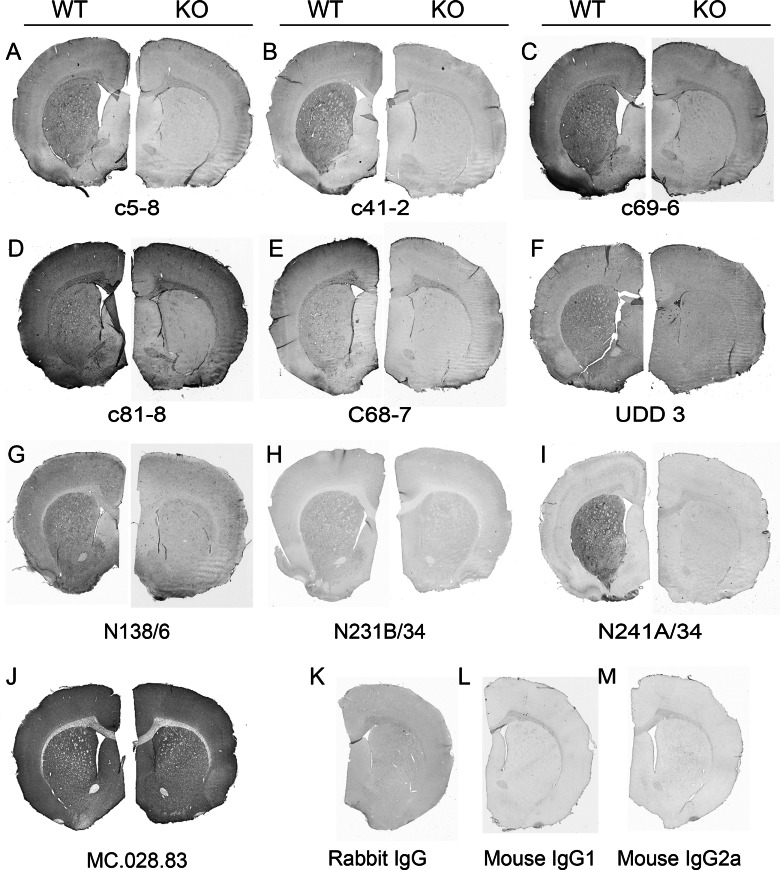
Anti-LRRK2 antibody immunoreactivity in rat brain (**A**–**J**) Summary of immunostaining for each antibody tested in WT and *LRRK2*-KO rat brain using PFA-perfused sucrose-preserved brain sections, stained free-floating. Dilutions in μg/ml were as follows: c5-8 (0.05), c41-2 and c69-6 (0.025), c81-8 (0.1), c68-7 (0.5), UDD 3 (0.001), N138/6 (0.4), N231B/34 (5.0), N241A/34 (0.25) and MC.028.83 (0.1). (**K**–**M**) Representative isotype controls for each serotype.

Following on from our optimized mouse brain free-floating protocol, as described above, we applied identical conditions to staining rat brain sections, with the only modification being that rat adsorbed anti-mouse secondary antibodies were used for mouse monoclonal antibody staining. Rat LRRK2 is very highly expressed in the striatum and less so in the cortex, in contrast with mouse LRRK2 that shows a more uniform distribution in both the striatum and cortical mantle. Rabbit monoclonal antibodies c5-8, c41-2 and c69-6 produced similar staining patterns (Figures 6A–6C), which were mostly consistent with distributions identified in mouse brain. Antibodies c81-8 and c68-7 did not produce specific staining (Figures 6D–6E). The rabbit monoclonal antibodies produced significant white matter staining in both WT and *LRRK2*-KO tissue; however, this was also observed in the IgG and no primary controls, albeit to a lesser extent. With the exception of c41-2, the rabbit monoclonal antibodies also produced significant non-specific glial staining in several areas of the brain, which morphologically resembled astrocytes (results not shown). The UDD 3 antibody did produce some specific staining in the striatum, but the overall signal to noise ratio was weak. The rabbit IgG and mouse IgG isotype controls had relatively weak overall colouration in contrast with darker signal that was observed in mouse brain tissue with these controls.

The best mouse monoclonal antibody staining in rat tissue was obtained with the N241A/34 antibody, which produced no significant staining in the *LRRK2*-KO animals under the conditions described (Figure 6I). At the level of the striatum, the immunostaining for N241A/34 and c41-2 in WT rat brain was identical. N138/6 and N231B/34 immunostaining did show some patterns consistent with the striatum and cortex, although the staining was confounded by heavy blood vessel staining. For MC.028.83, the signal to noise ratio between WT and *LRRK2* KO was weak.

Our recommendation for immunohistochemistry in rat free-floating rat brains is N241A/34, which produced staining of superior quality, with c41-2 being the second, but still highly recommended, choice.

### Comparison of LRRK2 reactivity in rodent cortex, striatum and Purkinje cells

**Figure 7:**
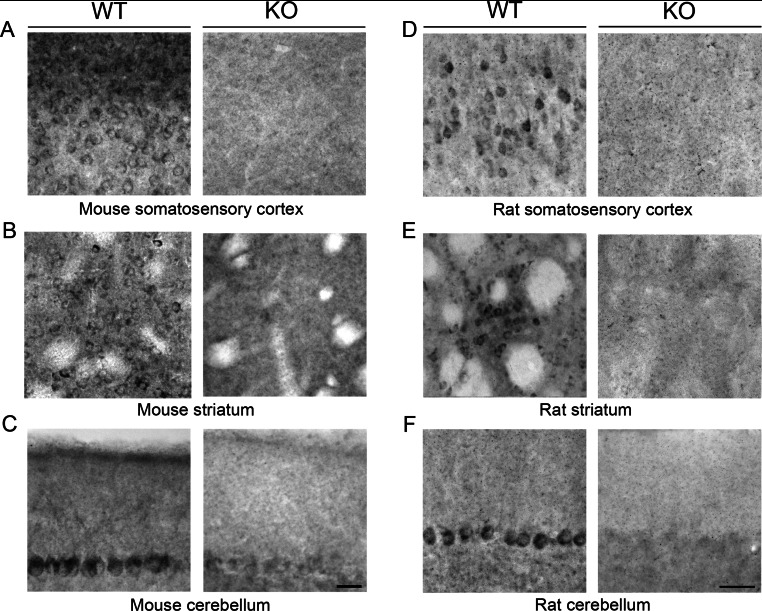
High-magnification images of LRRK2 immunoreactivity in somatosensory cortex, striatum and cerebellum Images were taken from (**A**–**C**) mouse WT and *LRRK2*-KO brain sections stained with c41-2 antibody, and (**D**–**F**) rat WT and *LRRK2*-KO brain stained with N241A/34 antibody. Scale bars, 50 μm.

We identified the brain regions conserved between mice and rats that have the highest level of LRRK2 expression. Representative areas of the cerebral cortex, the striatum and the cerebellar Purkinje layer are shown at higher magnification in Figure 7. Using the c41-2 antibody, we noted that cortical distribution is more refined in rats to lower layer pyramidal neurons (Figure 7D) as compared with mice, where expression was spread across the cortical layers. In the striatum, LRRK2 distribution was more confined to regions consistent with striosomes in rats as compared with more even distribution across the striatum in mice (Figures 7B and 7E). Overall, the most intense staining on an individual cell basis was observed in Purkinje neurons, although some background in these cells were observed in the KO and IgG control animal equivalents (Figures 7C and 7F; results not shown for IgG).

### Characterization of monoclonal antibody reactivity in human brain

#### Immunoblotting

**Figure 8:**
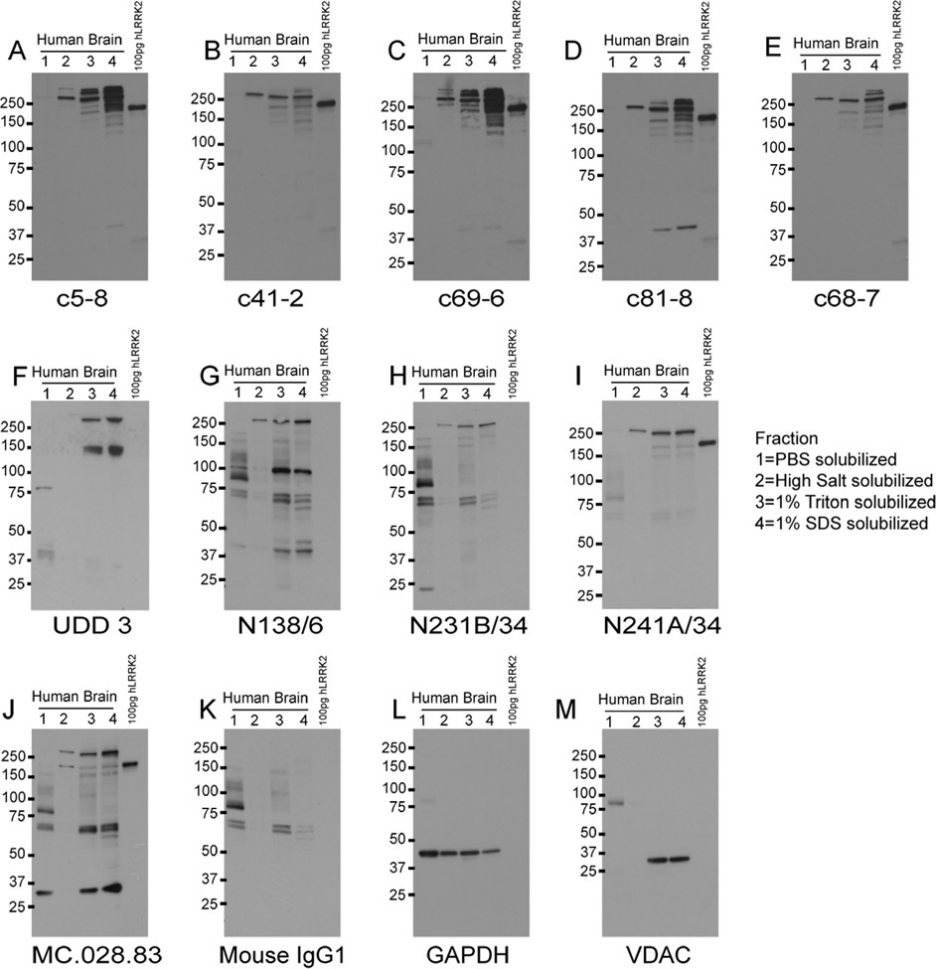
Summary blots of all anti-LRRK2 antibodies tested in human brain lysates from a series of differential extractions carried out in equivalent volumes Equal volumes were also loaded on to each gel. A total of 100 pg of human WT LRRK2 (amino acids 970–2527) recombinant protein was loaded as a control. All Epitomics and NeuroMab antibodies were used at 1 μg/ml, with the exception of UDD 3 which was used at 0.1 μg/ml, MC.028.83 antibody was used at 0.1 μg/ml. Molecular masses are indicated in kDa.

In human brain lysates, the Epitomics rabbit monoclonal antibodies all indicated a similar fractional distribution pattern for LRRK2, characterized by low LRRK2 concentrations in the PBS-soluble fraction and higher signal intensity with increasing buffer stringency (Figure 8). High-salt buffer was required for LRRK2 extraction, and although a single LRRK2 band was observed in the high-salt fraction for c41-2, c81-8 and c68-7, an additional higher-molecular-mass band was apparent in the detergent-soluble fractions. Thus this higher-molecular-mass off-target is both a relatively insoluble protein and conserved in mouse, rats and humans. Off-target proteins were also identified in all fractions for c5-8 and c68-6. The UDD 3 antibody intensely cross-reacted with a species near 150 kDa, and this species was also noted in rat and mouse brain tissue. The most efficacious antibody was mouse monoclonal antibody N241A/34 that detected a single band both in the high-salt and detergent fractions. LRRK2-sized bands were also observed for the other mouse monoclonal antibodies and a similar pattern of signal intensity to insolubility was noted. However, significant IgG cross-reactivity at lower molecular masses was observed even when using secondary antibodies pre-absorbed against human serum.

Overall, the mouse monoclonal antibody N241A/34 produced the most specific results by Western blot analysis and is the recommendation for human brain tissue lysates.

#### Immunohistochemistry

We performed a comprehensive immunostaining study in human post-mortem brain tissue sections, with testing of different staining conditions for both paraffin-embedded and fixed-frozen tissues. Of multiple regions tested, the cerebellum produced staining with a robust signal to noise ratio (Supplementary Figure S4 at http://www.biochemj.org/bj/453/bj4530101add.htm). The strongest staining was obtained with the UDD 3 antibody, which appeared to label the Bergmann glia that surrounds the Purkinje neurons. However, we were unable to convincingly demonstrate similar staining with the other antibodies. We thus advise UDD 3 staining to be interpreted with caution, given the presence of an additional band in the human tissue immunoblots (Figure 8F).

## DISCUSSION

There is an obvious need to have dependable, renewable and specific antibodies for biochemical analysis of proteins. LRRK2 has become a focus of intense research because of strong genetic associations with PD. Moreover, evolving data suggest that LRRK2 represents a kinase critical to disease pathogenicity, thus therapeutic efforts to develop LRRK2 inhibitors have been prioritized. Recently, ten new commercial monoclonal antibodies have been released, eight of which were generated with support from MJFF. These renewable resources should be critical in deciphering the role of LRRK2 in PD pathogenesis and as a therapeutic target for neuroprotection. However, individual laboratories are likely to be underpowered to perform the studies necessary to comprehensively validate each antibody in commonly used techniques. Indeed, the general sentiment in the field is that LRRK2 has proved refractory in the past to the development of standard and reliable protocols for biochemical and immunohistochemical analysis. MJFF assembled a group of investigators with the requisite expertise to perform these analyses utilizing all ten commercially available LRRK2 antibodies. The resulting standardized protocols are expected to aid investigators field-wide as they pursue research into LRRK2.

In the present study, we have used these ten monoclonal antibodies to develop the most robust and reliable protocols for immunoblotting detection, enzymatic assays and immunohistochemical localization in rodent brain tissue. Our results have emphasized the difficulty in developing protocols to detect LRRK2 in human post-mortem tissues and in dissociated neurons. A summary of our findings is presented in Table 1. With the exception of human lysates and brain sections, each experiment was controlled by tissue or cells derived from rat or mouse *LRRK2*-KO animals. Although all of the antibodies tested were able to recognize recombinant human LRRK2 purified from both bacterial and mammalian cell sources, there were differences in the human/mouse/rat species affinity, specificity and sensitivity (illustrated best in Figure 1). There was no single antibody identified that was suitable for every approach in every species. In fact, notable examples of cross-reactive targets that may otherwise confound future studies include the tendency of those rabbit monoclonal antibodies targeting an epitope in proximity of the LRR (leucine-rich repeat) domain to detect an additional higher-molecular-mass band in *LRRK2*-KO mouse material in detergent-lysed tissue, the rabbit monoclonal antibodies to detect glia in *LRRK2*-KO rats, the MC.028.83 antibody to detect intracellular filaments in *LRRK2*-KO mouse neurons, and the UDD 3 antibody to detect a band of size identical with that of LRRK2 via immunoblot in *LRRK2*-KO rats. However, this does not necessarily preclude these antibodies from use in other applications. For example, rabbit monoclonal c68-7 picks up the high-molecular-mass band in mouse cell lines, but is useful and specific in mouse brain lysate and in kinase assays. Notably, WT LRRK2 protein precipitated with c68-7 has greater than 5-fold activity over the *LRRK2*-KO and control incubations.

**Table 1:** Summary of recommended antibodies for selection with individual experimental applications to detect LRRK2 in mouse, rat and human tissues IP, immunoprecipitation with kinase assay; ICC, immunocytochemistry in hippocampal neurons; IHC-PFA, immunohistochemistry in PFA-perfused rodents. ✓, suitable; **×**, unsuitable; ∼, use with caution.

	Immunoblot			IP activity		ICC	IHC-PFA	
Antibody	Mouse	Rat	Human	Mouse	Human	Mouse	Mouse	Rat
c5-8	**×**	**×**	**×**	∼	∼	∼	∼	∼
c41-2	✓	**×**	∼	∼	∼	**×**	✓	∼
c69-6	**×**	**×**	**×**	∼	∼	∼	∼	∼
c81-8	**×**	**×**	**×**	∼	∼	∼	∼	**×**
c68-7	✓	**×**	∼	✓	✓	**×**	∼	**×**
UDD 3	∼	**×**	∼	✓	∼	**×**	**×**	∼
N138/6	∼	∼	**×**	**×**	**×**	**×**	**×**	∼
N231B/34	**×**	✓	∼	**×**	✓	∼	**×**	**×**
N241A/34	∼	✓	✓	**×**	✓	∼	**×**	✓
MC.028.83	**×**	**×**	**×**	∼	∼	**×**	**×**	**×**

Our comparison study has revealed some interesting insights into the differences between LRRK2 solubility in cell lines, rodent and human brain tissue. All but two antibodies were able to detect human lymphoblast lysed in a low-salt/Triton-based buffer. However, only one antibody (N241A/34) had the relative sensitivity to detect soluble LRRK2 in human brain lysate. On the other hand, in the high-salt lysate, most antibodies (except for c69-6, UDD 3 and MC.028.83) detected a single band. Inclusion of detergent in human tissue lysate revealed that only N231B/34 and N241A/34 were able to detect LRRK2 as the major band, whereas all others had considerable cross-reactivity with proteins of other sizes.

The ability of antibodies to efficiently immunoprecipitate LRRK2 from tissue or cells is a critical aspect to their use. The ability to detect very low amounts of protein and to capture active enzyme allow researchers to fully investigate the biochemistry of LRRK2 under conditions of interest. Our initial studies in HEK-293T cells overexpressing human LRRK2 identified the six most effective antibodies at immunoprecipitating LRRK2, which were then used to study the activity of immunoprecipitated LRRK2 from both human and mouse tissue and cells. Although previous studies have shown LRRK2 to be active against the substrate Nictide when immunoprecipitated from RAW264.7 macrophages [13], to our knowledge, the present study is the first to successfully purify active and endogenous LRRK2 from post-mortem brain tissue. All of the antibodies selected were able to immunoprecipitate active LRRK2 from human material, and most from mouse material. Background activity was low, as was evident by precipitations from *LRRK2*-KO tissue or with pre-immune experiments.

Until now, there has been limited information in the literature concerning antibody suitability in tissue sections, principally due to the lack of *LRRK2*-KO controls. Our comparison study does confirm previous reports of high expression in the striatum and cortex of rodents, but we note some differences between mice and rats, most notably that mice express LRRK2 at high levels throughout the cortex, with enrichment in the superficial cortical layers, whereas in rats, LRRK2 expression is much lower, with the strongest signal tending to be in the pyramidal layer. In mouse, the cortex and the striatum appear to be equally immunoreactive, but, in rats, the striatum is distinctly labelled in comparison with the adjacent cortex.

In summary, we present findings from a comprehensive and systematic study of ten new monoclonal antibodies. We have identified species-suitable antibodies for each application tested and we provide standard protocols that can be implemented in most laboratories. We also provide some insights into LRRK2 biology including its differential species solubility and expression patterns, which we envisage will open up a novel avenue for LRRK2 research.

## Online data

Supplementary data

## References

[1] Ross O. A., Soto-Ortolaza A. I., Heckman M. G., Aasly J. O., Abahuni N., Annesi G., Bacon J. A., Bardien S., Bozi M., Brice A. (2011). Association of *LRRK2* exonic variants with susceptibility to Parkinson's disease: a case-control study. Lancet Neurol..

[2] Daniels V., Baekelandt V., Taymans J. M. (2011). On the road to leucine-rich repeat kinase 2 signalling: evidence from cellular and *in vivo* studies. Neurosignals.

[3] Yue Z. (2012). Genetic mouse models for understanding LRRK2 biology, pathology and pre-clinical application. Parkinsonism Relat. Disord..

[4] West A. B., Moore D. J., Biskup S., Bugayenko A., Smith W. W., Ross C. A., Dawson V. L., Dawson T. M. (2005). Parkinson's disease-associated mutations in leucine-rich repeat kinase 2 augment kinase activity. Proc. Natl. Acad. Sci. U.S.A..

[5] Sheng Z., Zhang S., Bustos D., Kleinheinz T., Le Pichon C. E., Dominguez S. L., Solanoy H. O., Drummond J., Zhang X., Ding X. (2012). Ser^1292^ autophosphorylation is an indicator of LRRK2 kinase activity and contributes to the cellular effects of PD mutations. Sci. Transl. Med..

[6] Biskup S., Moore D. J., Celsi F., Higashi S., West A. B., Andrabi S. A., Kurkinen K., Yu S. W., Savitt J. M., Waldvogel H. J. (2006). Localization of LRRK2 to membranous and vesicular structures in mammalian brain. Ann. Neurol..

[7] Biskup S., Moore D. J., Rea A., Lorenz-Deperieux B., Coombes C. E., Dawson V. L., Dawson T. M., West A. B. (2007). Dynamic and redundant regulation of LRRK2 and LRRK1 expression. BMC Neurosci..

[8] Melrose H. L., Kent C. B., Taylor J. P., Dachsel J. C., Hinkle K. M., Lincoln S. J., Mok S. S., Culvenor J. G., Masters C. L., Tyndall G. M. (2007). A comparative analysis of leucine-rich repeat kinase 2 (Lrrk2) expression in mouse brain and Lewy body disease. Neuroscience.

[9] Lin X., Parisiadou L., Gu X. L., Wang L., Shim H., Sun L., Xie C., Long C. X., Yang W. J., Ding J. (2009). Leucine-rich repeat kinase 2 regulates the progression of neuropathology induced by Parkinson's-disease-related mutant α-synuclein. Neuron.

[10] Hinkle K. M., Yue M., Behrouz B., Dachsel J. C., Lincoln S. J., Bowles E. E., Beevers J. E., Dugger B. N., Kent C. B., Winner B. (2012). *LRRK2* knockout mice have an intact dopaminergic system but display alterations in exploratory and motor co-ordination behaviors. Mol. Neurodegener..

[11] Reed S. E., Staley E. M., Mayginnes J. P., Pintel D. J., Tullis G. E. (2006). Transfection of mammalian cells using linear polyethylenimine is a simple and effective means of producing recombinant adeno-associated virus vectors. J. Virol. Methods.

[12] Bradford M. M. (1976). A rapid and sensitive method for the quantitation of microgram quantities of protein utilizing the principle of protein-dye binding. Anal. Biochem..

[13] Nichols R. J., Dzamko N., Hutti J. E., Cantley L. C., Deak M., Moran J., Bamborough P., Reith A. D., Alessi D. R. (2009). Substrate specificity and inhibitors of LRRK2, a protein kinase mutated in Parkinson's disease. Biochem. J..

[14] Deng X., Dzamko N., Prescott A., Davies P., Liu Q., Yang Q., Lee J. D., Patricelli M. P., Nomanbhoy T. K., Alessi D. R., Gray N. S. (2011). Characterization of a selective inhibitor of the Parkinson's disease kinase LRRK2. Nat. Chem. Biol..

[15] Moehle M. S., Webber P. J., Tse T., Sukar N., Standaert D. G., DeSilva T. M., Cowell R. M., West A. B. (2012). LRRK2 inhibition attenuates microglial inflammatory responses. J. Neurosci..

[16] Higashi S., Moore D. J., Colebrooke R. E., Biskup S., Dawson V. L., Arai H., Dawson T. M., Emson P. C. (2007). Expression and localization of Parkinson's disease-associated leucine-rich repeat kinase 2 in the mouse brain. J. Neurochem..

[17] Melrose H., Lincoln S., Tyndall G., Dickson D., Farrer M. (2006). Anatomical localization of leucine-rich repeat kinase 2 in mouse brain. Neuroscience.

[18] Taymans J. M., Van den Haute C., Baekelandt V. (2006). Distribution of PINK1 and LRRK2 in rat and mouse brain. J. Neurochem..

