# Supplementary material for: Comprehensive characterization and optimization of anti-LRRK2 (leucine-rich repeat kinase 2) monoclonal antibodies
Source: Biochem J. 2013 Jun 13;453(Pt 1):101–13. doi: 10.1042/BJ20121742 (PMC3682752; doi:10.1042/BJ20121742)
Supplement: Supplementary data [file bj4530101add.pdf]

BJ Disease

Biochem. J. (2013) **453**, 101–113 (Printed in Great Britain) doi:10.1042/BJ20121742

online data

SUPPLEMENTARY ONLINE DATA

Comprehensive characterization and optimization of anti-LRRK2 (leucine-rich repeat kinase 2) monoclonal antibodies

Paul DAVIES*[1], Kelly M. HINKLE†[1], Nour N. SUKAR‡[1], Bryan SEPULVEDA§, Roxana MESIAS§, Geidy SERRANO||, Dario R. ALESSI*, Thomas G. BEACH||, Deanna L. BENSON§, Charles L. WHITE, III¶, Rita M. COWELL**, Sonal S. DAS††, Andrew B. WEST‡[2] and Heather L. MELROSE†[2,3]

The Michael J. Fox LRRK2 Monoclonal Antibody Consortium: *MRC Protein Phosphorylation Unit, University of Dundee, Nethergate, Dundee DD1 4HN, Scotland, U.K., †Department of Neuroscience, Mayo Clinic Jacksonville, 4500 San Pablo Road, Jacksonville, FL 32224, U.S.A., ‡Department of Neurology, University of Alabama at Birmingham, 1530 3rd Ave S, Birmingham, AL 35294, U.S.A., §Department of Neuroscience, Mount Sinai School of Medicine, 1425 Madison Ave, New York, NY 10029, U.S.A., ||Civin Laboratory for Neuropathology, Banner Sun Health Research Institute, 10515 West Santa Fe Drive, Sun City, AZ 85351, U.S.A., ¶Department of Pathology, Division of Neuropathology, University of Texas Southwestern Medical Center, 5323 Harry Hines Boulevard, Dallas, TX 75390, U.S.A., **Department of Psychiatry, University of Alabama at Birmingham, 1530 3rd Ave S, Birmingham, AL 35294, U.S.A., and ††The Michael J. Fox Foundation for Parkinson's Research, Grand Central Station, P.O. Box 4777, New York, NY 10163-4777, U.S.A.

IMMUNOCYTOCHEMISTRY IN DISSOCIATED PRIMARY NEURONS

Hippocampi were dissected from postnatal day 1–2 WT and *LRRK2*-KO mice [1], cells were dissociated in 0.1 % papain and plated on coverslips coated with poly-L-lysine and laminin (Sigma) at a density of 1.4×10^4 cells/cm^2. Neurons were maintained in Neurobasal Media (Invitrogen) containing NS21 supplements [2]. Neurons were cultured for 10 days, then fixed for immunostaining. Four different conditions were tested. (1) 4 % PFA + 4 % sucrose, 15 min at 37 °C and permeabilization with 0.25 % Triton X-100 for 5 min, all in PBS at pH 7.2. (2) Fixation as above followed by antigen retrieval: 10 mM sodium citrate (pH 6.0) for 5 min in microwave at 665W [3], followed by permeabilization as in (1). (3) 2 % PFA + 0.2 % glutaraldehyde in PBS for 15 min at 37 °C and permeabilization as in (1). (4) Fixation and permeabilization with methanol for 10 min at −20 °C. By comparing labelling in WT and *LRRK2*-KO tissue, the conditions tested could be ranked: $1 \geqslant 3 > 2 = 4$. Non-specific binding was blocked by incubation in 10 % BSA in PBS for 1 h at room temperature. Primary antibodies were diluted in 1 % (w/v) BSA to the appropriate concentration and incubated for 16 h at 4 °C. To visualize labelling, neurons were incubated in DyLight 488-conjugated anti-mouse or anti-rabbit secondary antibodies (Jackson ImmunoResearch Laboratories) and covered with a coverslip using Mowiol. As a negative control, neurons were incubated in mouse or rabbit IgG, followed by secondary antibody as indicated above. Images were acquired on a Zeiss 510 laser-scanning confocal microscope using a ×100 objective.

IMMUNOHISTOCHEMISTRY WITH FFPE (FORMALIN-FIXED PARAFFIN-EMBEDDED) TISSUE

For FFPE from UTSW, brains removed at autopsy were fixed whole by immersion in 20 % neutral-buffered formalin at room temperature for at least 10 days. Fixed tissue was dehydrated and embedded in paraffin using standard procedures. Sections were cut at 4 μm. Immunostaining was performed on a Leica Bond III automated immunostaining platform. Endogenous peroxidase was inhibited with 3–4 % H_2O_2. All primary incubations were carried out at room temperature for 15–30 min. All antibodies and development reagents were dissolved in Tris-buffered saline. Washing between steps (4–6 min) was carried out using Tris-buffered saline. A heat-induced epitope-retrieval technique was used, treating tissue sections in an EDTA-based heat-induced epitope-retrieval buffer (pH 8.9–9.1) (Bond Epitope Retrieval Solution 2, Leica) at 100 °C for 30 min on the Leica Bond III instrument (on a mini-hotplate-type heating block) before application of primary antibody. All sections received standardized signal development steps utilizing a proprietary multivalent detection kit (Bond Polymer Refine Detection, Leica) containing an unlabelled rabbit secondary antibody raised against mouse IgG and a goat secondary antibody raised against rabbit IgG and tagged with a multivalent (mouse + rabbit) secondary antibody–polymer–horseradish peroxidase conjugate, with DAB as the chromogen and haematoxylin counterstain. When mouse tissue sections were stained with primary antibodies raised in rabbit, the rabbit anti-mouse secondary antibody was eliminated in order to minimize non-specific background staining in mouse tissue. Paraffin sections from mouse *LRRK2*-KO brain [4] were run as negative control sections.

An analogous procedure was developed at Banner Sun Health Research Institute in order to adapt the UTSW procedure, which was performed with some agents that are proprietary and primarily applicable to users of Leica immunostainers, to agents that are all commercially available to the general scientific community. The modifications included replacement with the proprietary heat-induced epitope-retrieval EDTA buffer with Tris/EDTA buffer and replacement of the proprietary multivalent secondary antibody–polymer–horseradish peroxidase complex with an ABC horseradish peroxidase complex and DAB enhanced with nickel ammonium sulfate. Sections were pre-treated by heating to 95 °C in 1 mM sodium EDTA/10 mM Tris buffer (final pH 9.0) for 30 min in a water bath, then successively incubated with 1 % H_2O_2 to suppress endogenous peroxidase (30 min), primary antibody (UDD 3 or N241A/34), secondary antibody (Vector Laboratories, biotinylated anti-rabbit or anti-mouse IgG, 1:1000 dilution), ABC reagent (Vector Laboratories; 1:1000 dilution) and DAB saturated with nickel ammonium sulfate and imidazole. Sections were washed between steps in three 5-min changes of buffer. All solutions except for DAB (0.05 M Tris buffer, pH 7.6) were made up with 0.1 M PBS (pH 7.4) with 0.3 % Triton X-100.

[1] These authors contributed equally to this work.
[2] To whom correspondence should be addressed (email abwest@uab.edu or melrose.heather@mayo.edu).
[3] Heather Melrose has received intellectual property royalties for *LRRK2*-knockout mice.

© The Authors Journal compilation © 2013 Biochemical Society

Biochemical Journal www.biochemj.org

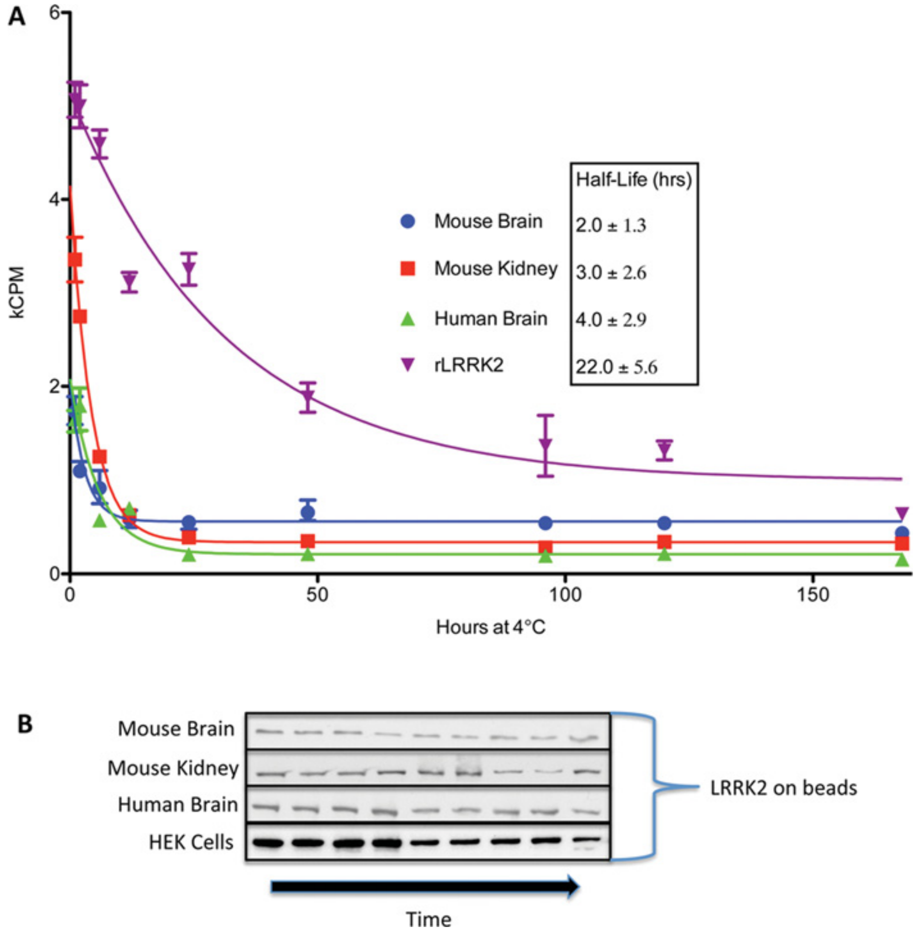

Figure S1 Assessment of the effect of 'post-mortem' interval on the activity of LRRK2 immunoprecipitated from human and mouse tissue and incubated on ice for the indicated time

A control immunoprecipitation from HEK-293T cells overexpressing wild-type LRRK2 before the incubation is included. All precipitations were carried out with UDD 3. Relative activity is assessed as c.p.m. from ^{32}P decay of bound and labelled ATP. Statistical analysis using a two-way ANOVA revealed significant effects ($P < 0.0001$) for both time and lysate source. All blots were probed with UDD 3 antibody at 0.1 μg/ml and show the LRRK2 on the IP (immunoprecipitation) beads following the kinase assay. Recorded activities were normalized against the LRRK2 IP input, as determined by Western blotting. rLRRK2, recombinant LRRK2.

© The Authors Journal compilation © 2013 Biochemical Society

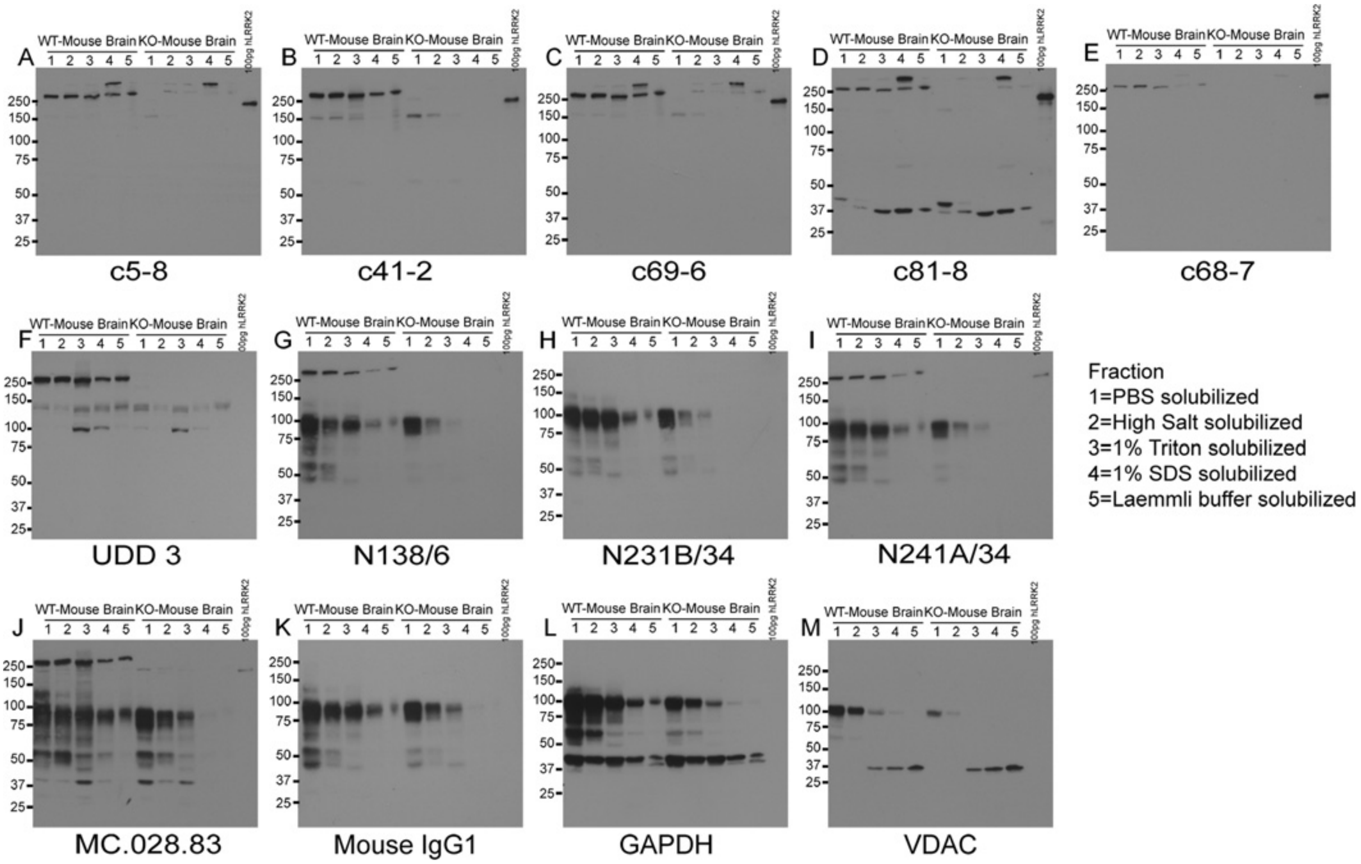

Figure S2 Summary blots of all anti-LRRK2 antibodies tested in WT and *LRRK2*-KO mouse brain lysates from a series of differential extractions carried out in equivalent volumes

Equal volumes were also loaded on to each gel. A total of 100 pg of human WT LRRK2 (amino acids 970–2527) recombinant protein was loaded as a control. All Epitomics and NeuroMab antibodies were used at 1 μg/ml, with the exception of UDD 3 which was used at 0.1 μg/ml, MC.028.83 antibody was used at 0.1 μg/ml concentration. Molecular masses are indicated in kDa.

© The Authors Journal compilation © 2013 Biochemical Society

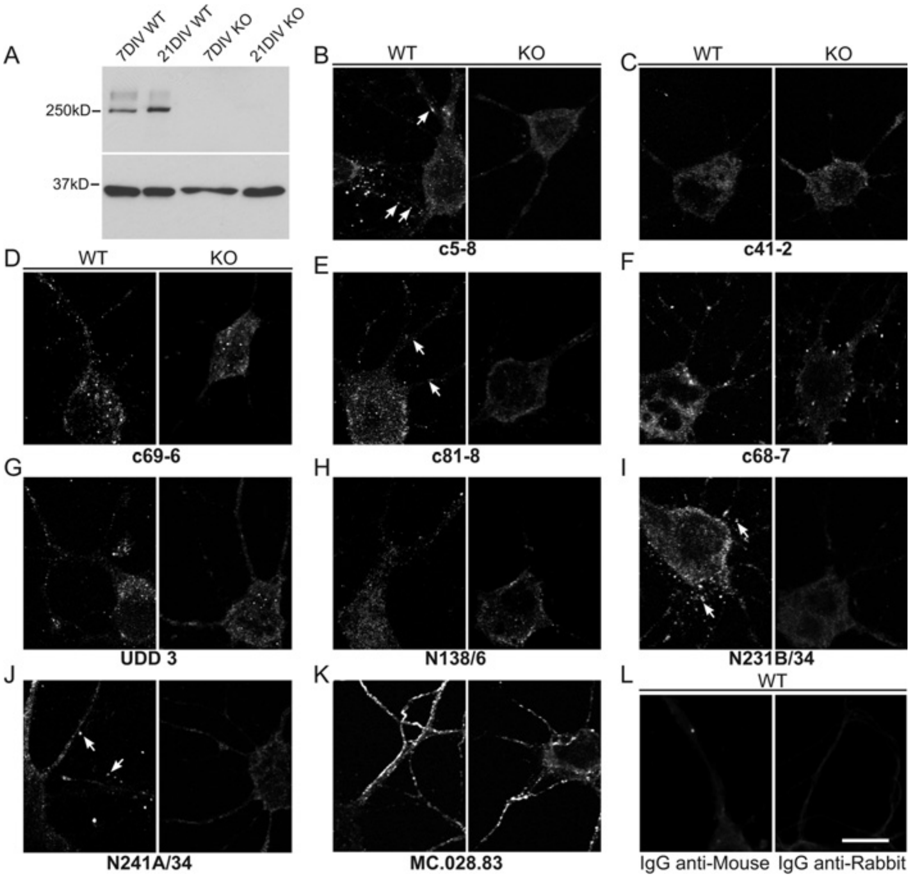

Figure S3 Summary of LRRK2 presence and distribution in hippocampal neurons grown in culture

Immunoblot for LRRK2 (**A**, upper panel) shows a prominent 250 kDa band at 7 DIV (days *in vitro*) that is more intense at 21 DIV in lysates from WT neurons and absent from *LRRK2*-KO neurons. GAPDH (**A**, lower panel) was used as a loading control. Molecular masses are indicated in kDa. (**B**–**L**) Confocal images show immunolocalization for the anti-LRRK2 antibodies (**B**–**K**) or IgG controls (**L**) indicated in WT or *LRRK2*-KO neurons fixed with 4 % PFA and permeabilized with 0.25 % Triton X-100. Examples of LRRK2 labelled intracellular clusters are indicated by arrows and also serve to indicate the antibodies showing the most consistent labelling patterns (**B**, **E**, **I** and **J**). WT neurons incubated with mouse or rabbit IgG before secondary labelling as a control (**L**). Scale bar, 10 μm.

© The Authors Journal compilation © 2013 Biochemical Society

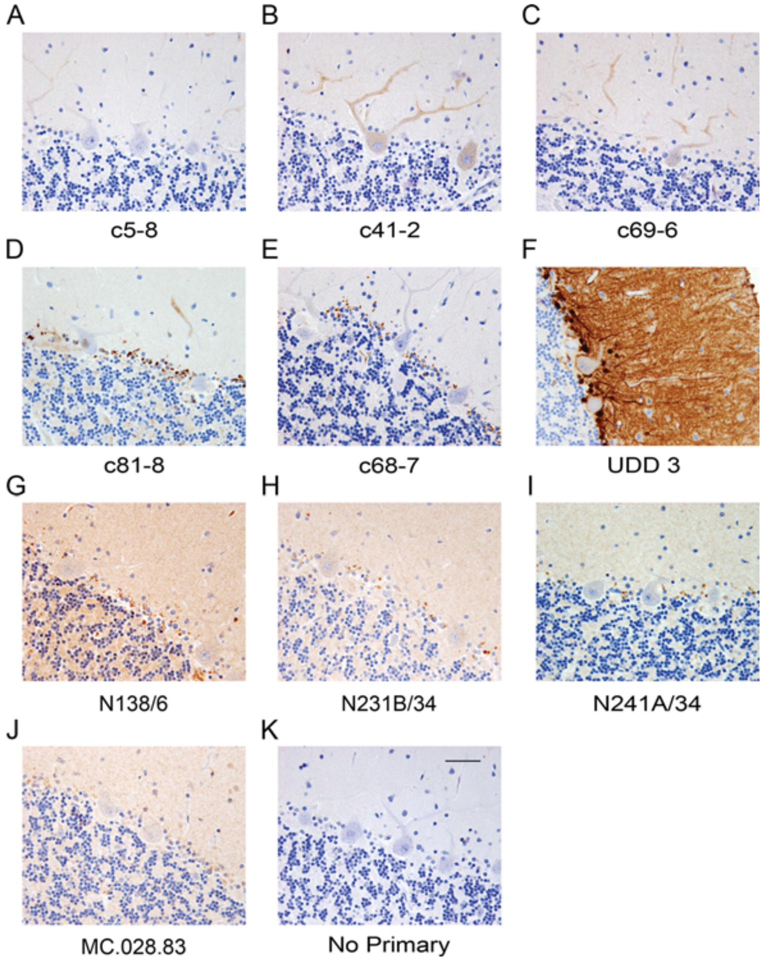

Figure S4 Anti-LRRK2 antibody immunoreactivity in human brain

(**A**–**J**) Summary of immunostaining for each antibody tested in human cerebellum using formalin-fixed paraffin-embedded sections. Dilutions in μg/ml were as follows: c5-8 (20), c41-2 (0.5), c69-6 (2), c81-8 (4), c68-7 (40), UDD 3 (0.5), N138/6 (5), N231/B/34 (10), N241A/34 (2.5) and MC.028.83 (0.625). (**K**) Representative no primary control. Scale bar, 50 μm.

REFERENCES

1 Lin, X., Parisiadou, L., Gu, X. L., Wang, L., Shim, H., Sun, L., Xie, C., Long, C. X., Yang, W. J., Ding, J. et al. (2009) Leucine-rich repeat kinase 2 regulates the progression of neuropathology induced by Parkinson's-disease-related mutant α-synuclein. Neuron **64**, 807–827

2 Chen, Y., Stevens, B., Chang, J., Milbrandt, J., Barres, B. A. and Hell, J. W. (2008) NS21: re-defined and modified supplement B27 for neuronal cultures. J. Neurosci. Methods **171**, 239–247

3 Martins, A. R., Dias, M. M., Vasconcelos, T. M., Caldo, H., Costa, M. C., Chimelli, L. and Larson, R. E. (1999) Microwave-stimulated recovery of myosin-V immunoreactivity from formalin-fixed, paraffin-embedded human CNS. J. Neurosci. Methods **92**, 25–29

4 Hinkle, K. M., Yue, M., Behrouz, B., Dachsel, J. C., Lincoln, S. J., Bowles, E. E., Beevers, J. E., Dugger, B. N., Kent, C. B., Winner, B. et al. (2012) *LRRK2* knockout mice have an intact dopaminergic system but display alterations in exploratory and motor co-ordination behaviors. Mol. Neurodegener. **7**, 25

Received 19 November 2012/18 March 2013; accepted 5 April 2013
Published as BJ Immediate Publication 5 April 2013, doi:10.1042/BJ20121742

© The Authors Journal compilation © 2013 Biochemical Society
